# Gut microbiota shape the inflammatory response in mice with an epithelial defect

**DOI:** 10.1080/19490976.2021.1887720

**Published:** 2021-02-28

**Authors:** Ran Wang, Md Moniruzzaman, Kuan Yau Wong, Percival Wiid, Alexa Harding, Rabina Giri, Wendy (Hui) Tong, Jackie Creagh, Jakob Begun, Michael A. McGuckin, Sumaira Z. Hasnain

**Affiliations:** a Immunopathology Group, Mater Research Institute – The University of Queensland, Translational Research Institute, Brisbane, Australia; b Inflammatory Bowel Disease Group, Mater Research Institute - The University of Queensland, Translational Research Institute, Brisbane, Australia; c Mater Adult Hospital, Mater Health Services, South Brisbane, Australia; d Faculty of Medicine, Dentistry and Health Sciences, The University of Melbourne, Australia; e Australian Infectious Disease Research Centre, The University of Queensland, Brisbane, Australia

**Keywords:** Microbiota, epithelial cells, Germ free, Inflammation, mucin

## Abstract

Intestinal epithelial cell endoplasmic reticulum (ER) stress has been implicated in intestinal inflammation. It remains unclear whether ER stress is an initiator of or a response to inflammation. *Winnie* mice, carrying a *Muc2* gene mutation resulting in intestinal goblet cell ER stress, develop spontaneous colitis with a depleted mucus barrier and increased bacterial translocation. This study aims to determine whether the microbiota was required for the development of *Winnie* colitis, and whether protein misfolding itself can initiate inflammation directly in absence of the microbiota. To assess the role of microbiota in driving *Winnie* colitis, *WT* and *Winnie* mice on the same background were rederived into the germ-free facility and housed in the Trexler-type soft-sided isolators. The colitis phenotype of these mice was assessed and compared to *WT* and *Winnie* mice housed within a specific pathogen-free facility. We found that *Winnie* colitis was substantially reduced but not abolished under germ-free conditions. Expression of inflammatory cytokine genes was reduced but several chemokines remained elevated in absence of microbiota. Concomitantly, ER stress was also diminished, although mucin misfolding persisted. RNA-Seq revealed that *Winnie* differentiated colon organoids have decreased expression of the negative regulators of the inflammatory response compared to *WT*. This data along with the increase in *Mip2a* chemokine expression, suggests that the epithelial cells in the *Winnie* mice are more responsive to stimuli. Moreover, the data demonstrate that intestinal epithelial intrinsic protein misfolding can prime an inflammatory response without initiating the unfolded protein response in the absence of the microbiota. However, the microbiota is necessary for the amplification of colitis in *Winnie* mice. Genetic predisposition to mucin misfolding in secretory cells initiates mild inflammatory signals. However, the inflammatory signal sets a forward-feeding cycle establishing progressive inflammation in the presence of microbiota.

**Abbreviations:** Endoplasmic Reticulum: ER; Mucin-2: Muc-2; GF: Germ-Free; Inflammatory Bowel Disease: IBD

## Introduction

Inflammatory bowel diseases (IBD), including Crohn’s disease (CD) and ulcerative colitis (UC), are chronic inflammatory disorders with multiple contributing factors. Accumulating evidence demonstrates that dysregulation or altered balance between composition of the intestinal microbiota, intestinal epithelial integrity, and mucosal immune responses can trigger IBD. Among all three compartments, epithelial integrity is particularly important as it is a primary target of inflammation and also contributes to the initiation of inflammation.^1^ Studies in mouse models of UC highlight the importance of the protective mucus barrier and the role of mucin-producing goblet cells in regulating barrier function. Defects in mucin glycosylation and the major intestinal mucin Muc2 result in increased experimental colitis severity.^2–5^ *Winnie* mice with single missense mutation in the *Muc2* mucin gene have depleted goblet cells and a diminished mucus layer with increased epithelial permeability, and intestinal inflammation akin to ulcerative colitis.^6^ *Winnie* colitis is characterized by a T_H_17 immune phenotype involving both innate and adaptive immunity.^7^ This mouse model provides important evidence that protein misfolding in secretory cells and associated endoplasmic reticulum (ER) stress and can be a primary trigger of colitis. ER stress and the unfolded protein response (UPR) that alleviates protein misfolding and restores ER homeostasis, can also drive intestinal inflammation.^8–10^ This potentially sets up a forward feeding cycle of ER stress and inflammation because the UPR leads to immune activation via multiple pathways and, in turn, inflammatory cytokines, and reactive oxygen species (ROS) produced by immune cells exacerbate ER stress (these relationships are reviewed in^11^). Importantly, *Winnie* mice respond to anti-inflammatory drugs that are used clinically to treat patients with UC, including glucocorticoids^12^ and thiopurines.^13^ In both cases, suppression of *Winnie* colitis is accompanied by reduced ER stress and UPR activation, less Muc2 misfolding, restoration of mature mucin production, and amelioration of goblet cell depletion. While glucocorticoids have a co-contributing direct effect on goblet cell oxidative and ER stress, thiopurines had no direct effects on secretory cell ER stress. These data are consistent with inflammation exacerbating Muc2 misfolding and ER stress.

Intestinal microbiota play an essential role in regulating epithelial function and maintaining intestinal homeostasis, however in most settings microbes are required for intestinal inflammation. Mice carrying genetic defects predisposing to colitis that are born and raised in a germ-free (GF) environment without exposure to any live microbes generally fail to develop intestinal inflammation.^14^ Studies in GF mice have also shown that microbiota-dependent signals regulate mucus production by goblet cells and epithelial integrity.^1,15^ Consistent with the requirement for microbes to trigger inflammation, upon DSS challenge, GF mice only had minimal inflammatory response, but somewhat paradoxically these mice suffered enhanced epithelial injury and mortality as a consequence of a weakened intestinal barrier and direct effects of DSS on the epithelium.^16^

Although both secretory cell ER stress and gut microbiota dysregulation can initiate inflammation, the relative contribution of these two parameters in driving colitis progression remains unknown. It is also unclear whether protein misfolding associated ER stress itself can directly trigger inflammation in the absence of microbiota, and whether inflammation influences the degree of ER stress experienced by epithelial cells, particularly in the cells that undertake substantial biosynthesis of proteins in the secretory pathway. In this study, we have used the *Winnie* spontaneous colitis model to address these questions demonstrating that in the absence of the microbiota, epithelial intrinsic protein misfolding initiates inflammation via downregulation of the anti-inflammatory regulators. Gut microbiota is required to fully activate and exacerbate inflammation however, protein misfolding still persists in the absence of microbes.

## Results

### Reduced histological colitis in Winnie mice raised under germ-free (GF) conditions

**Figure 1.**
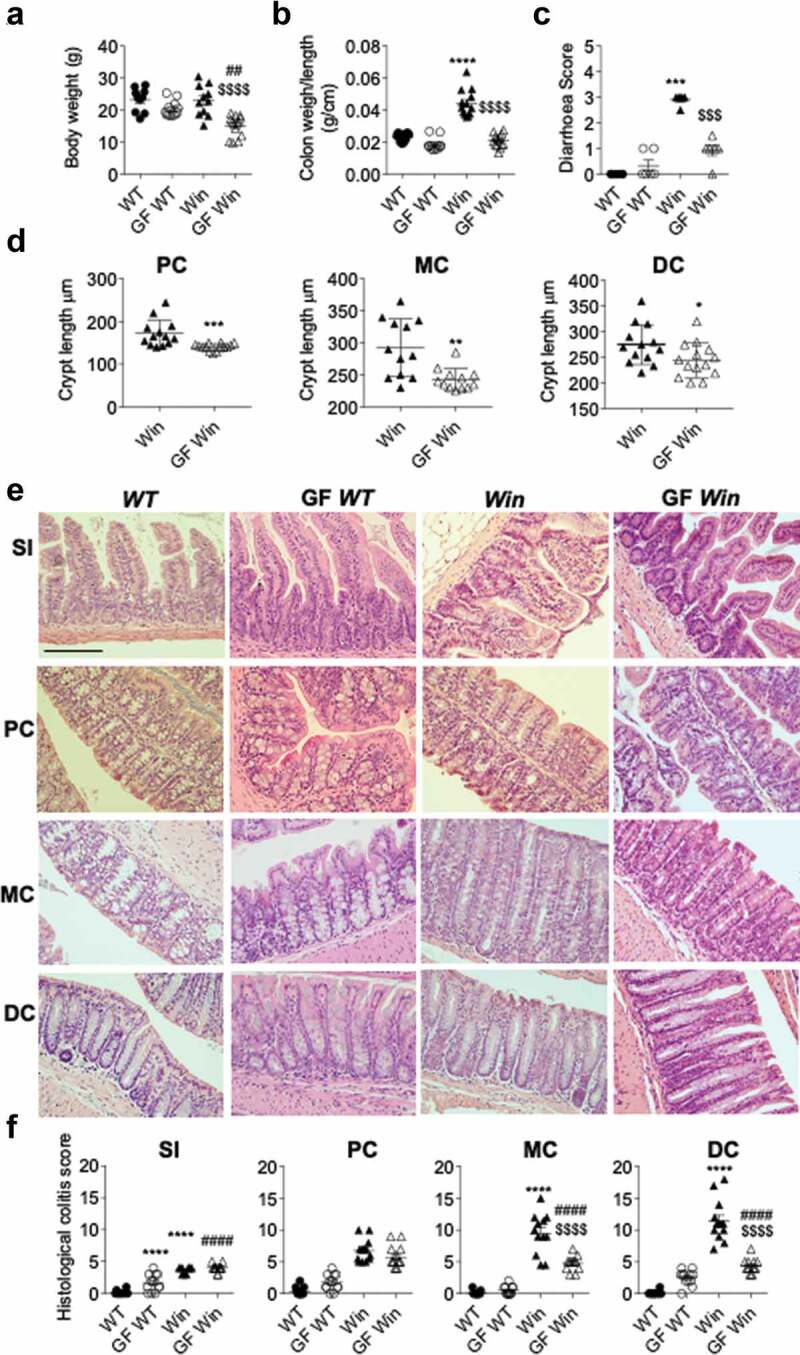
Absence of microbiota alleviated colitis in *Winnie* mice. *WT*, GF *WT, Winnie* and GF *Winnie* mice were sacrificed at 6–8 weeks of age. (a), Body weight. (b), Colon weight to length ratio. (c), Diarrhea score. (d), Crypt length in all mice was measured microscopically and presented as average crypt length per mouse in proximal (PC), middle (MC) and distal colon (DC) regions. (e), Representative H&E-stained small intestine and colon sections. (f), Blind assessment of histological colitis of mice in abased on H&E stained sections (maximum score = 20). Values are expressed as mean ± SEM and individual data points, n = 10 to 14. a, b, cand f, One-way ANOVA with Bonferroni’s multiple comparison test; *compared to *WT* control, #compared to GF *WT*, and $compared to *Win* (**P < .01; ***P < .001; ****P < .0001). Scale bar = 200 µm

To determine the role of the microbiota in the development of intestinal inflammation, we re-derived male *Winnie* mice into a GF facility and compared them to male *Winnie* mice housed in a conventional specific-pathogen-free facility. Age-matched male GF *C57BL/6* (*WT*) mice from the same facilities were used as controls. 6–8 week old GF *Winnie* mice had lower body weight than conventional *Winnie* mice or GF *WT* or conventional *WT* mice (Figure 1a). Colon weight and diarrhea scores of GF *Winnie* were reduced compared to *Winnie* mice, suggesting *Winnie* spontaneous colitis is substantially reduced in the absence of the microbiota (Figure 1b,c). No major changes were observed with microbiota depletion in the histological appearance of the small intestine and proximal colon of *Winnie* mice where the inflammation is mild (Figure 1e-f). However, while the absence of microbiota did not alter the colonic histology of *WT* mice, it substantially reduced histological colitis in the mid and distal colonic regions of *Winnie* mice where colitis is most severe (Figure 1e-f). Reduced crypt length in all regions of colon (Figure 1d), improved crypt structure and decreased inflammatory cell infiltration (Figure 1e) contributed to reduced histological scores in GF *Winnie* compared to *Winnie* mice (Figure 1f).

### Reduced colonic inflammation and ER stress in Winnie mice in the absence of the microbiota

**Figure 2.**
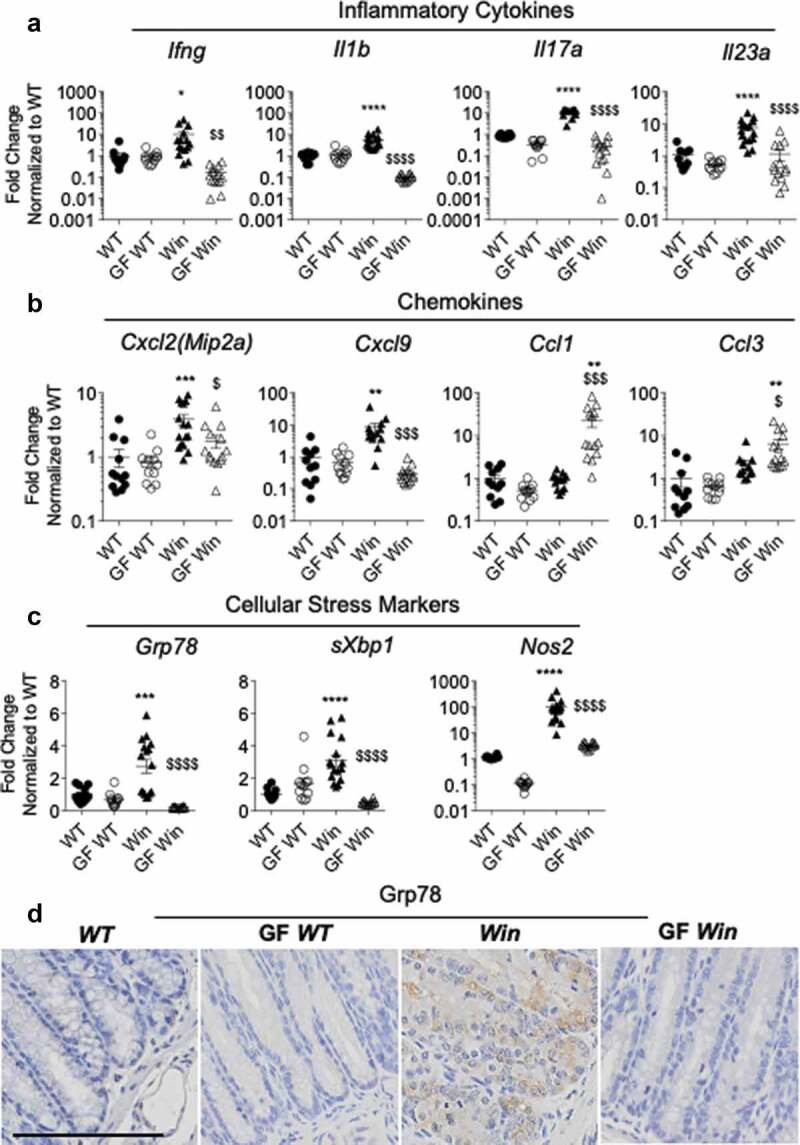
Inflammation and ER stress were reduced in the distal colon of GF *Winnie*. (a-c): Distal colonic mRNA expression of genes encoding inflammatory cytokines (a), intestinal epithelial-specific chemokines (b), and cellular stress markers (c) in mice described in Figure 1. (d), Representative immunohistochemistry with Grp78 antibody reflective of ER stress in distal colon sections. (a-c): Data presented as fold change corrected to *b actin* and normalized to *WT* mice. Values are expressed as mean ± SEM and individual data points, n = 10 to 14. One-way ANOVA with Bonferroni’s multiple comparison test; *compared to *WT* control, #compared to GF *WT*, and $compared to *Win* (*P < .05; **P < .01; ***P < .001; ****P < .0001)

The intestinal inflammation of *Winnie* mice progresses with prominent distal colitis featuring elevated expression of inflammatory cytokines and chemokines as well as increased ER stress.^6,7^ mRNA expression of multiple inflammatory effector cytokines was decreased in the distal colon of GF *Winnie* compared to *Winnie* mice (Figure 2a). *Ifng, Il1b, Il17a* and *Il23a* mRNA levels decreased to the levels comparable to *WT* mice. In *WT* mice cytokine gene expression was unaffected by microbiota status. In contrast, variation was seen in the response of chemokine genes to GF conditions in *Winnie* mice. While the chemokines, *Cxcl2* (encodes Mip2α) and *Cxcl9* were downregulated in GF *Winnie* compared to *Winnie* mice, in contrast, *Ccl1* and *Ccl3* (encodes Mip1α) were up-regulated in GF *Winnie* mice compared to *Winnie* mice.

Next, we sought to determine whether ER stress was alleviated because of the altered intestinal inflammation in GF *Winnie* mice. Using qRT-PCR and immunohistochemistry, we found lower levels of ER stress and oxidative stress markers in the absence of microbiota. Expression of *Grp78, sXbp1*, and *Nos2* was increased in distal colon of *Winnie* mice compared to *WT* mice, and lower in the absence of microbiota to levels seen in *WT* mice (Figure 2c). Confirming the mRNA data, immunohistochemistry showed the chaperone Grp78, that is elevated during protein misfolding and ER stress, accumulated in the ER region of colonic goblet cells of *Winnie* mice, but was not seen in GF *Winnie* mice (Figure 2d). These data show that reduced ER stress in GF *Winnie* mice occurs concomitantly with reduced inflammation, suggesting that in conventional conditions inflammation exacerbates protein misfolding and ER stress.

### Reduced Intestinal Epithelial Proliferation and Restored Paneth and Goblet Cell Secretion in Winnie Mice in the Germ-Free Environment

Epithelial hyperproliferation is a key feature of colitis in *Winnie* mice. To determine the proliferation rate of crypt epithelial cells, we performed immunohistochemistry staining for Ki67. The number of Ki67-positive proliferating cells were lower in small intestine of GF *WT* compared to *WT*, and also in the distal colon of the GF *Winnie* mice compared to *Winnie* mice (Supplemental Figure 1a-d), consistent with previously reported decreased epithelial turnover in the absence of luminal microbes.^17^ *Winnie* mice had increased proliferation in the distal colon (Supplemental Figure 1d) consistent with an inflammation-driven proliferative response associated with crypt elongation, as previously described,^6^ and under GF conditions proliferation reduced to near levels seen in conventional *WT* mice though greater than in GF *WT* mice  (Figure 2b).

Reduced mucin volume in the theca of goblet cells and reduced Paneth cell granule formation are also key features of *Winnie* colitis. The expression of Paneth cell-derived anti-microbial peptides has been shown to be modulated and dependent on, the presence of signals from the microbiota.^18^ Lysozyme immunohistochemistry revealed that in conventional conditions Paneth cells in *Winnie* mice possess fewer secretory granules compared to *WT* mice due to chronic ER stress in these Muc2-producing cells (Supplemental Figure 1e). In addition, both *WT* and *Winnie* mice housed in GF conditions had reduced number of secretory granules in Paneth cells compared to their counterparts raised in the conventional environment, consistent with previous findings that the microbiota stimulates anti-microbial peptide production (Supplemental Figure 1f). mRNA expression of genes encoding Paneth cell signature anti-microbial peptides *Reg3b* and *Reg3g* (Supplemental Figure 1e) mirrored the decrease in lysozyme granule immunohistochemistry in GF conditions in both *WT* and *Winnie* mice (Supplemental Figure 1f-g).

**Figure 3.**
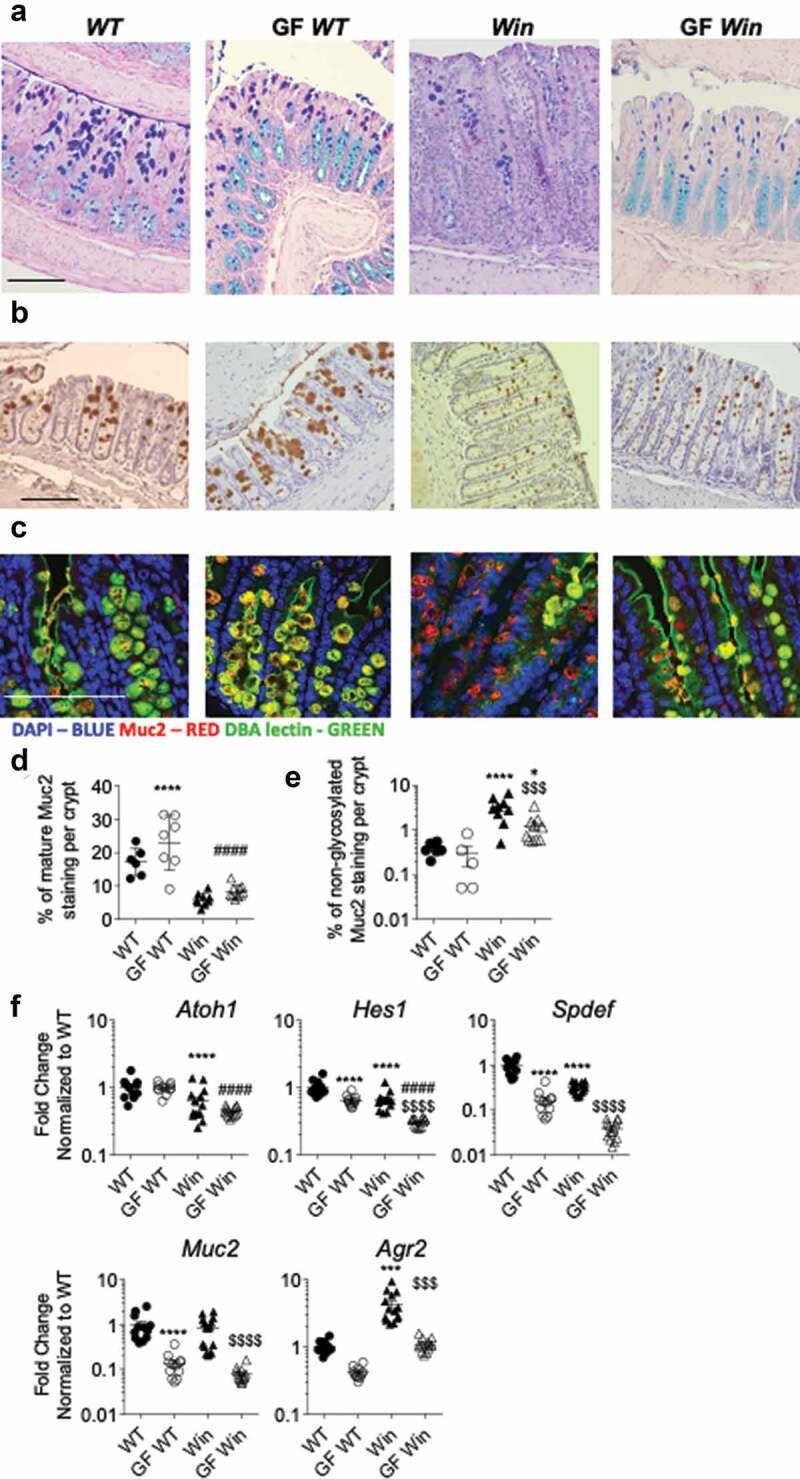
Muc2 misfolding and goblet cell mucin storage were partially improved in the distal colon of GF *Winnie* mice. (a), Representative sections of Alcian blue and periodic-acid Schiff stain, (b) immunohistochemistry with mature Muc2 antibody showing goblet cell morphology and (c) immunofluorescent staining of Muc2 core peptide (Red) and DBA lectin O-glycan staining (Green) showing accumulation of non-glycosylated misfolded Muc2 precursor in the distal part of the colon of mice described in Figure 1. D, Quantification of total Muc2 staining in the distal colon. (e), Quantification of non-glycosylated Muc2 staining (misfolded Muc2 in Red) in the distal colon. (f), mRNA expression of *Atoh1*(Math1), *Hes1, Spdef, Muc2 and Agr2* in the distal colon of mice in Figure 1. Values are expressed as mean ± SEM and individual data points, n = 10 to 14, mixed gender. One-way ANOVA with Bonferroni’s multiple comparison test; *compared to *WT* control, #compared to GF *WT*, and $compared to *Win* (***P < .001; ****P < .0001). Scale bar = 200 µm

Microbiota colonization and TLR/NOD ligands have been shown to regulate mucin gene expression and promote mucin secretion from goblet cells.^19^ To further elucidate the complex relationship between ER stress, inflammation, and mucin production in intestinal epithelial cells, we examined colonic goblet cell morphology and function in GF *WT* and *Winnie* mice. Under GF conditions *Muc2* mRNA levels decreased ~10-fold in both *WT* and *Winnie* mice, consistent with the previous finding^17^ that diminished secretion of Muc2 (and therefore a decreased requirement for biosynthesis) is potentially as a result of lacking stimulation from bacterial products including TLR/NOD ligands (Figure 3c-f). In *WT* mice, GF conditions were accompanied by little change (if anything a slight increase) in the size of the goblet cell theca representing stored mucin granules for secretion (Figure 3a-d). As previously published,^6^ *Winnie* mice showed the characteristic small goblet cell theca in distal (Figure 3a-c) as well as proximal colon and small intestine (Supplemental Figure 2). The *Winnie* mice goblet cell theca size slightly increased under GF conditions (Figure 3a-d). Co-staining with the DBA lectin which binds O-glycans added in the Golgi apparatus and a Muc2 peptide antibody demonstrated the accumulation of the unglycosylated misfolded Muc2 precursor in *Winnie* goblet cells (red staining in Figure 3c). Under GF conditions the accumulation of Muc2 precursor was reduced (Figure 3e). Moreover, mRNA expression of the secretory cell lineage-determining transcription factor *Atoh1* was not altered while enterocyte lineage transcription factor *Hes1* expression was lower in both *WT* and *Winnie* under GF conditions (Figure 3f). Expression of transcription factor *Spdef* which drives goblet cell secretory function was lower in *Winnie* mice probably reflecting premature death of stressed goblet cells, however its target gene *Agr2* was strongly induced in *Winnie*, which may reflect upregulation of this ER protein disulfide isomerase by the UPR. *Spdef* expression decreased substantially in *WT* and *Winnie* mice under GF conditions, and consequently, the genes it drives, *Muc2* and *Agr2*, decreased similarly (Figure 3f), showing that the restored goblet cell theca could be a result of increased translation or reduced secretion, rather than a direct consequence of elevated *Muc2* transcription. Overall, this result indicates that intestinal bacterial colonization promotes intestinal epithelial cell proliferation, goblet cell mucin production, and Paneth cell secretory function. In *Winnie* mice carrying a *Muc2* misfolding mutation, the increased mucin production driven by bacterial colonization results in greater Muc2 misfolding, ER stress, and goblet cell depletion.

### Muc2 misfolding initiates inflammation directly via downregulation of the endogenous anti-inflammatory regulators in epithelial cells

**Figure 4.**
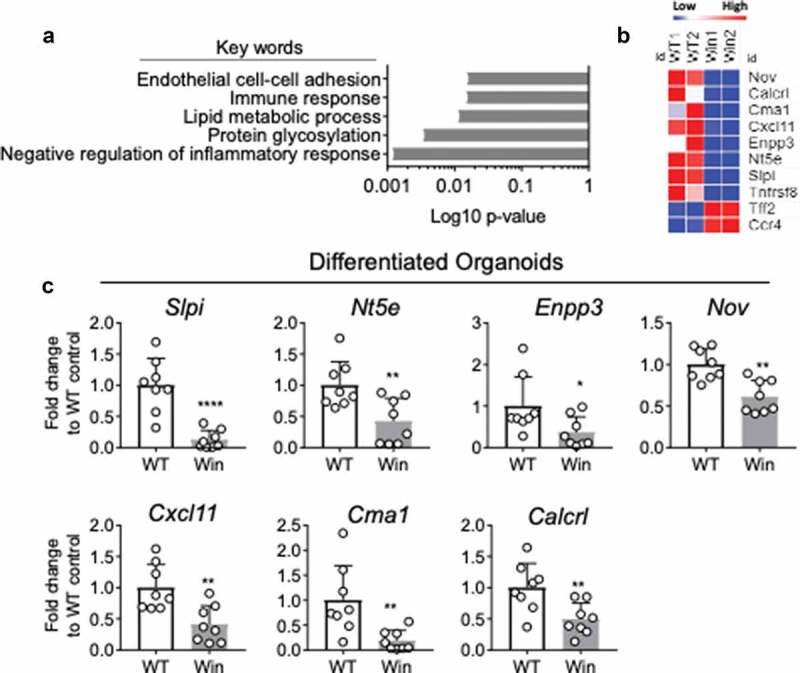
RNA-sequencing reveals lower expression of negative regulators of inflammation in differentiated *Winnie* compare to *WT* organoids. (a), DAVID gene ontology analysis reveals top five pathways/keywords associated with the 119 mostly differentially expressed genes between differentiated *Winnie* and *WT* organoids. (b), Heatmap showing expression levels of genes in “Negative regulation of inflammatory response”. (c), mRNA expression of *Slpi, Nt5e, Enpp3, Nov, Cxcl11, Cma1* and *Calcrl* in a separate set of colon organoids isolated from *WT and Winnie* mice and differentiated with DAPT for 48 h. n = 8 in C per group, each dot represents organoid isolated from one mouse. Mann-Whitney test (*P < .05)

In GF *Winnie* mice, expression of ER stress and UPR markers were downregulated to a similar level as *WT* GF mice (Figure 2c,d). Intestinal inflammation was significantly reduced but not abolished in GF *Winnie* mice, mucin misfolding was reduced, and molecular markers of ER stress and activation of the UPR returned to levels seen in *WT* mice. Together, this suggests that Muc2 misfolding can initiate inflammatory signals directly, thus initiating inflammatory pathology when microbes are present. To test this hypothesis, colonic crypts were isolated from both *WT* and *Winnie* mice and cultured *in vitro* to remove the epithelium from the influence of microbial products and non-epithelial immune factors. Morphologically, organoids from *Winnie* mice were larger with slightly less budding observed compare to those from *WT* mice (Supplemental Figure 3a). Colonic organoids were next cultured and differentiated in presence of a Notch γ-secretase inhibitor (DAPT). This condition primarily promotes goblet cell differentiation and mucin biosynthesis, making it a preferred method to assess Muc2 protein misfolding associated functional changes. The differentiation was confirmed by the upregulation of *Muc2* mRNA expression (Supplemental Figure 3b). Using RNA-Seq the differences in transcriptome between differentiated *WT* and *Winnie* epithelial cells were investigated 48 h after differentiation. We found that amongst all genes that are differentially regulated between *WT* and *Winnie*, most genes were downregulated in *Winnie* compare to *WT* organoids (Supplemental Figure 4a). An RNA-Seq signature comprising of 119 genes was generated based on greater than two-fold enriched with FDR of <0.01 in *WT* organoids compared to *Winnie* organoids. Pathway analysis using DAVID Bioinformatics was performed and the most significant keywords associated with the 119 gene signature were found including “negative regulation of inflammatory response” and “immune response” (Figure 4a and Supplemental Figure 4b). A number of genes known to negatively regulate inflammatory responses including *Slpi, Nov, Calcrl, Cma1, Cxcl11, Tnfrsf8, Nt5e* and *Enpp3* were downregulated in differentiated *Winnie* compared to *WT* organoids (Figure 4b). We validated this result using quantitative RT-PCR in a separate set of differentiated *WT* and *Winnie* organoids for all genes (Figure 4c), except for *Tnfrsf8* whose expression trended downward but was not significant (Supplemental Figure 4c). Concurrently, chemokine receptor *Ccr4* and the trefoil factor (TFF) family member, spasmolytic peptide (SP)/*Tff2* were upregulated, although these genes could not be confirmed in a second experiment using qRT-PCR validation (Supplemental Figure 4c). The downregulation of genes associated with negative regulation of inflammatory responses in *Winnie* compared to *WT* organoids suggests the ability of *Winnie* epithelial cells to prime a highly regulated tolerogenic immune response under sterile conditions is impaired.

**Figure 5.**
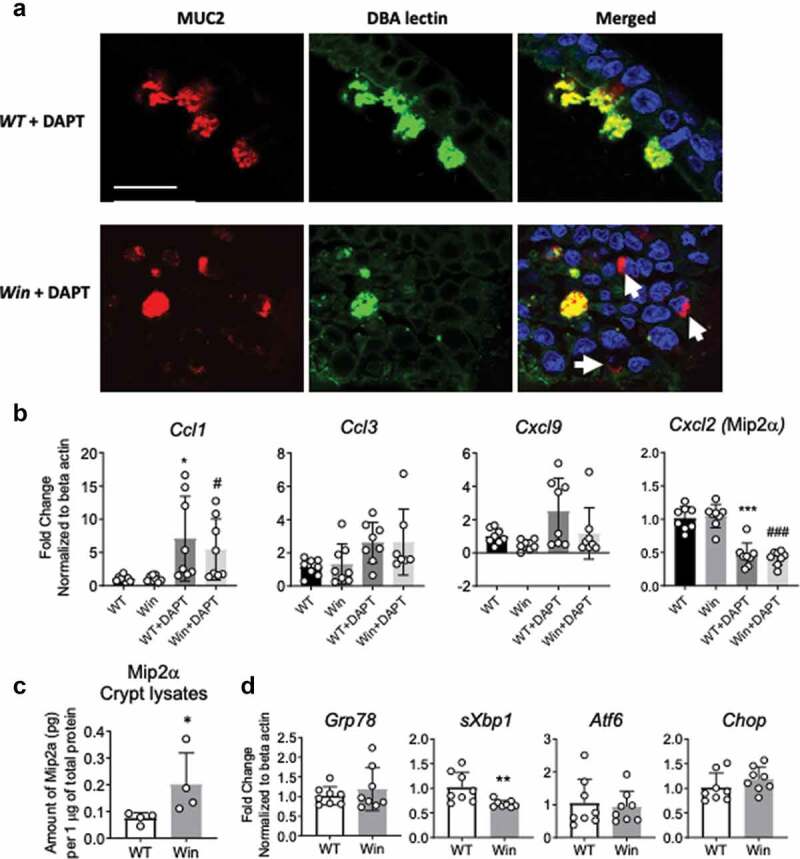
Muc2 misfolding persists in *in vitro* differentiated colonic organoids resulting in altered chemokine expression in epithelial cells. (a), Immunofluorescent staining of Muc2 core peptide (Red) and DBA lectin O-glycan staining (Green) showing Muc2 precursor accumulating in the non-glycosylated form in the DAPT differentiated *Winnie* organoids compared to *WT*. (b), mRNA expression of chemokines *Ccl1, Ccl3,Cxcl9* and *Mip2a* in the organoids in *(a)*. (c), Levels of Mip2a protein in colonic crypt isolated from whole colon of 8 weeks old *WT* and *Winnie* mice raised in conventional condition. (d), mRNA expression of ER stress and UPR markers in organoids in *(a)*. For b and d, each dot indicates organoid isolated from one mouse, n = 8 per group, values are expressed as mean ± SD and individual data points: For C, each dot indicates one mouse, n = 4 per group. B, One-way ANOVA with Bonferroni’s multiple comparison test; *compared to *WT* control, #compared to *Winnie* (*P < .05; ** *P* < .01; ***P < .001). C and D, Mann-Whitney test, ns – not significant. Scale bar in A = 20 µm

*Winnie* spontaneous colitis originates from Muc2 misfolding associated ER stress and UPR activation, which further leads to impaired barrier integrity with increased bacteria translocation. To assess whether Muc2 misfolding itself is driving sterile inflammation via the induction of ER stress and UPR activation in *Winnie* epithelial cells, *WT* and *Winnie* colonic organoids were differentiated with DAPT for 48 h (promotes Muc2 biosynthesis and folding). DAPT increased Muc2 protein synthesis in both *WT* and *Winnie* colonic organoids, however Muc2 was not appropriately glycosylated (less co-localization of DBA lectin with Muc2) in *Winnie* compared to *WT* organoids (Figure 5a, lower magnification in Supplemental Figure 5a). This data suggests that *Muc2* mutation-induced protein misfolding occurs in *Winnie* organoids *in vitro*. Based on this, it was hypothesized that Muc2 misfolding could induce ER stress and initiate the UPR. Interestingly, gene expression of ER stress and UPR markers were similar or lower in differentiated *Winnie* organoids compared to *WT* organoids (Figure 5d), suggesting that Muc2 misfolding is not sufficient to drive UPR activation *in vitro* in absence of microbial and inflammatory factors in the environment. To address whether Muc2 misfolding itself triggered inflammatory signals, the same chemokines altered *in vivo* (Figure 2b) were measured by qPCR in organoids and by ELISA in culture supernatants (Figure 5b). Expression of *Ccl1* was increased with differentiation, while others including *Ccl3, Cxcl9* were unchanged (Figure 5b). Chemokines at protein level were undetectable in both differentiated *WT* and *Winnie* organoids culture supernatants, which may be due to the *in vitro* culture environment. To demonstrate the epithelial-specific chemokine involvement in *Winnie* colitis *in vivo*, colonic epithelial cells were isolated from *WT* and *Winnie* mice using an established protocol.^20^ Then, Mip2a protein was measured by ELISA in *ex vivo* isolated crypt lysates, then normalized to total protein quantity. Colonic epithelial cells in *Winnie* mice, primed by the *in vivo* microenvironment, in the presence of microbiota and immune cells, contained higher levels of Mip2a compared to *WT* mice (Figure 5c). Overall, our data show that mucin production can trigger subtle inflammatory signals directly at transcriptional level. In absence of a comprehensive anti-inflammatory network in *Winnie* epithelial cells, these signals become amplified *in vivo* in the presence of microbiota and inflammation.

## Discussion

**Figure 6.**
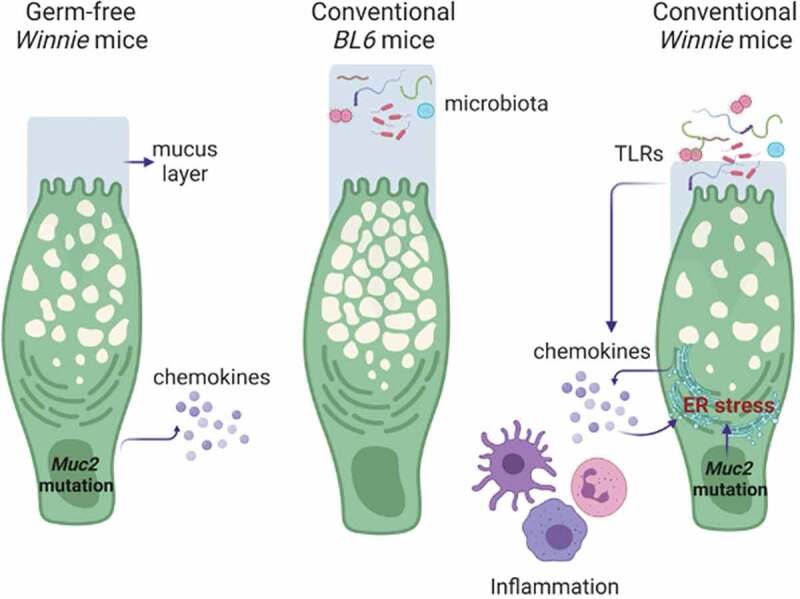
Complex relationship of epithelium defect, microbiome translocation and mucosal inflammation. Under a germ-free environment, Muc2 misfolding in epithelial cells initiate inflammatory signals directly, but the signal is only significantly amplified by the presence of gut microbiota and immune cells in a conventional environment. In turn, the inflammation and microbiota translocation (or TLR leakage) can exacerbate Muc2 misfolding and ER stress resulting in mucus layer depletion and increased bacteria translocation. This sets a feed-forward cycle for progressive intestinal inflammation in *Winnie* mice. Figure created using BioRender

In this study, we have determined the effects of the intestinal microbiota on spontaneous colitis development in a mouse model with an intrinsic goblet cell protein misfolding defect. By comparing mice in conventional and germ-free conditions, and by *in vitro* organoid culture we demonstrate that mucin misfolding in goblet cells is sufficient to condition epithelial cells to become more responsive to stimuli, but that the microbiota is required for the full-scale development of spontaneous colitis in *Winnie* mice. The reduced severity of colitis in GF conditions was characterized by reduced inflammatory cell infiltration and effector cytokine expression in the mucosa. Therefore, our results suggest that while protein misfolding and ER stress prime intestinal inflammation, the microbiota exacerbates inflammation and ER stress in this model and is required for the full disease phenotype, including diarrhea. Our findings are consistent with previous reports where mice housed under GF environment failed to develop intestinal inflammation in multiple experimental colitis models, including genetic deficiencies in IL-10 and IL-2, and the T cell transfer and chemically induced colitis models.^21–23^ The major reason for the reduced inflammation is likely due to the absence of microbiota driving activation of innate and adaptive immunity^24–26^ (Figure 6). One limitation of the current study is that *WT*and *Winnie* mice were not littermates and therefore could harbor different gut microbiota. However, we have compared *Winnie* mice to littermate control *WT* mice, and *Winnie* mice to *WT* mice ordered from a different animal facility, and the same colitis phenotype was consistently observed in *Winnie* mice.^6,20^ In addition, *Winnie* mice housed either under SPF or clean conventional conditions showed the same colitis phenotype in both housing conditions.^6^ A recent paper used 16S rRNA metagenetic analysis to study the gut microbiota composition of age-matched littermate *WT* and *Winnie* mice. It showed that dysbiosis in *Winnie* mice was already established at 4 weeks of age, before substantial histologic evidence of gut inflammatory changes, and these microbial communities diverged from that derived from their heterozygous mothers.^27^ Results from this study demonstrated that *Muc2* genetic mutation is involved in the complex regulation of intestinal homeostasis, such as promoting early gut dysbiosis that is independent from maternal microbial transfer.^27^ These results are consistent with our current findings that the *Muc2* mutation drives the colitis phenotype, while microbiota is the amplifier.

Muc2 protein misfolding and ER stress in intestinal secretory cells of *Winnie* mice can be exacerbated by inflammation.^6,7^ This is evident with anti-inflammatory drug treatment^12^ and depletion of T and B cells^7,28^ in the *Winnie* mice which alleviate both ER stress and colitis. It is important to note that anti-inflammatory drugs or biologics we have used including glucocorticoids,^12^ thioguanine^13^, and IL-23p19 antibody,^29^ not only suppressed inflammation, but also alleviated ER stress in *Winnie* mice providing direct evidence that inflammation exacerbates ER stress in this model. Although a mucin misfolding mutation is the primary driver of *Winnie* colitis, it is also recognized that secretory cells such as goblet and Paneth cells are targeted by inflammation.^28,30^ Accompanying reduced inflammation and ER stress, in the absence of microbiota, there was an increase in stored mucins in the goblet cells of GF *Winnie* when compared to *Winnie* mice. Goblet cell differentiation marker Math1 (*Atoh1*) is unchanged in GF compare to conventional conditions, but goblet cell markers including *Spdef* and *Muc2* gene expression were markedly reduced in GF mice compared to relative conventional raised mice, suggesting that microbiota regulates intestinal epithelial secretory function, which has been reported in multiple studies.^1,31^ Paneth cell production of anti-microbial molecules was also diminished under GF conditions compared to conventionally raised mice, confirming that microbiota regulates intestinal secretory function in both major intestinal secretory cell types.^32^

Although ER stress and UPR activation are involved in IBD pathogenesis,^33^ Muc2 misfolding did not induce measurable ER stress and UPR activation in *Winnie* mice housed under GF conditions. Expression of inflammatory chemokines were largely similar in differentiated *WT* and *Winnie* organoids. There was an increase in chemokine expression in the *Winnie* mice *in vivo*, suggesting that these changes are either lost due to the removal of inflammatory milieu in culture or the main source of chemokines seen *in vivo* are leukocytes. Additionally, *Mip2a* expression in organoids is decreased with DAPT differentiation, suggesting enterocytes, not goblet cells, are the major cellular source. These transcriptional changes of chemokines were too subtle to be detected at the protein level in organoid culture supernatants. When we look at the global transcriptome landscape, *Winnie* epithelial cells carrying *Muc2* mutation present a less immunosuppressive phenotype with lower expression of negative regulators of inflammation compared to *WT* epithelial cells. Among all immune suppressors, the most inhibited genes in *Winnie* compared to *WT* organoids was *Slpi* (secretory leukocyte protease inhibitor) – a well-known peptide protecting mucosal tissue against the detrimental consequences of inflammation.^34^ Another well-known negative regulator of inflammation is the adenosine pathway involving *Nt5e* (CD73) and *Enpp3* (ecto-pyrophosphatase/phosphodiesterase-3) which are ectoenzymes that control extracellular nucleotide and nucleoside levels. Both enzymes catalyze ATP to AMP, therefore driving a shift from an ATP-driven proinflammatory environment to an anti-inflammatory milieu induced by adenosine.^35,36^ Significantly decreased CD73 expression in *Winnie* epithelial cells compared to *WT* could result in high ATP, therefore, sustained inflammatory signals. This is intriguing, since ATP has been shown to be one of the damage-associated molecular patterns (DAMPs) in cancer cells causing immunogenic cell death.^37^ Although detailed mechanism of *Muc2* mutation driven downregulation of purinergic pathways is unknown, unfolded Muc2 in the ER could modify the cellular redox state and initiate inflammatory signals. Other negative regulators of inflammation include *Nov* also known as CCN3 – a matricellular protein that negatively regulates nuclear factor kappaB (NF-kappaB) activity;^38^ and *Cxcl11* expression drives CD4+ T cell polarization into IL-10-producing type 1 regulatory T cells.^39,40^ Genes like *Cma1* and *Calcrl* have not been reported to be expressed in epithelial cells, but their immune-modulatory roles are known in other settings. *Cma1* expression in immune cells (Mast cells) plays a role in maintaining intestinal barrier function and to expel parasitic worms.^41,42^ *Calcrl* encoding *c*alcitonin receptor-like receptor is important in maintaining lymphatic tissue homeostasis. Loss of *Calcrl* expression in lymphatic tissue exacerbates intestinal pathology following mucosal injury demonstrating the importance of lymphatic function in promoting recovery from intestinal inflammation.^43^ Taken together, our *in vitro* data in primary organoids highlights the important function of mucosal epithelial cells in priming a more inflammatory response to the luminal microbiota.

Defects in intestinal epithelial barrier function are a characteristic feature of human IBD. Although *Muc2* gene polymorphisms have not been associated with increased risk of IBD in GWAS, epithelial integrity is linked with IBD pathogenesis. Mucosal barrier perturbations can affect different levels of the protective mechanisms including alterations of PRRs, a defective mucus layer, alterations in the process of autophagy, or an increased epithelial barrier permeability, and thereby result in inadequate protection against microbial adherence and invasion. Like the *Winnie* mice, bacterial translocation due to impaired barrier function in humans results in chronic intestinal inflammation in IBD. Since IBD affects predominantly the most heavily colonized gut segments – terminal ileum and colon, an involvement of the microbiota in disease progression is obvious and supported by success of fecal microbiota transplants. Post-operative recurrence of CD is also triggered by luminal contents.^44^ Because the microbiota plays important roles in driving and amplifying chronic inflammation, it is reasonable to target the microbiota in order to dampen inflammation. Meta-analysis revealed that fecal transplantation could be a promising treatment of IBD patients.^45,46^ This approach can be an exciting opportunity for patients carrying genetic mutations predisposing them to increased risk of having IBD.

In the current study, we have shown that in absence of microbiota, intestinal inflammation, and epithelial ER stress are significantly alleviated in a Muc2 misfolding induced spontaneous colitis mouse model. Data from this study provide valuable information on how epithelial cell intrinsic defects, inflammation, and bacteria translocation inter-regulate each other in the complex intestinal mucosal system. This work explains how genetic predispositions to protein misfolding could combine with the microbiota to drive progressive barrier depletion and inflammation.

## Materials and methods

### Animals

Conventionally raised *Winnie* mice on a *C57BL/6* background (bred in-house) and *C57BL/6* mice (purchased from ARC) were housed within a specific pathogen-free Biological Research Facility (BRF) at the Translational Research Institute (TRI, free of *Norovirus* and *Helicobacter*) and fed autoclaved food. *C57BL/6* mice from ARC were acclimatized for 2 weeks in the TRI-BRF before used for experiments. *C57BL/6* and *Winnie* mice were rederived into the GF facility at Water and Eliza Hall Institute in Melbourne and housed in the trexler-type soft-sided isolators. For assessing the colitis phenotype of these mice, age and gender-matched (only male animals were used in this study). GF *C57BL/6* and *Winnie* mice were shipped to the TRI BRF at Brisbane in microisolator cages. All animal research and experiment protocols were approved by the Animal Ethics Committee of the University of Queensland and conducted according to National Health and Medical Research Council guidelines.

### Mouse tissue end-point collection

Upon arrival, diarrhea scoring was performed on all mice. The diarrhea scoring system can be interpreted as follows: 0 – no diarrhea: solid stool; 1 – very mild diarrhea: formed stools that appear moist on the outside; 2 – mild diarrhea: formed stools that easily submit to pressure applied with forceps; 3 – diarrhea: no fully formed stools with a mucous-like appearance; 4 – severe, watery diarrhea: mostly clear or mucous-like liquid stool with very minimal solid present. Then, GF *C57BL/6* and *Winnie* mice were sacrificed on the same day and colonic tissues were harvested for downstream analysis. The whole colon, the cecum and ileum (distal 1/3^rd^ part of the small intestine) were removed. Each segment of the intestine was opened longitudinally and the fecal content was gently removed. Colon weight was measured and then the colon was dissected longitudinally into two sections. One of the sections was rolled (Swiss rolled) from the rectum along with cecum and ileum and fixed in 10% neutral buffered formalin for histological examination. The other section of the colon was dissected in equal halves, designated as ‘proximal’ and ‘distal’ colon and snap frozen in dry ice for later mRNA expression analysis.

### Quantitative reverse-transcription polymerase chain reaction

Tissue samples (snap frozen and homogenized) were extracted into TRIzol before RNA purification using ISOLATE II RNA Mini Kit (Bioline, London, UK), followed by cDNA synthesis using SensiFAST™ cDNA Synthesis Kit (Bioline, London, UK) containing oligo (dT) and random hexamer primers. SensiFAST™ SYBR® Lo-ROX Kit (Bioline, London, UK) was used for quantitative polymerase chain reaction using a ViiA 7 (Life Technologies, Carlsbad, CA); passive reference dye ROX was used. Results were analyzed using ViiA 7 RUO software v1.2 (Applied Biosystems, Life Technology). Primer efficiencies were determined using complementary DNA and primer dilutions for each gene of interest. Primers used to determine gene expressions are summarized in Supplemental Table 1. All cytokine gene expression data were normalized to the housekeeping gene beta actin as a fold change compared with the average of the control group. Samples having non-detectable cytokine cDNA amplification were given an arbitrary Ct value of 37 representing the limit of detection.

### Hematoxylin & eosin staining

Formalin-fixed tissue samples were processed into paraffin blocks and sectioned by the TRI Histology Core Service. After de-waxing in xylene and re-hydrating through ethanol gradients (100%, 95%, 90% 70%, and dH_2_O), paraffin sections were placed in Hematoxylin reagent (Gill’s formula, No.2, Sigma-Aldrich) for 30 s and then washed under running tap water for 5 min. Slides were then placed in Eosin Y reagent (Sigma-Aldrich) for 30 s. After Eosin staining, slides were dehydrated in 90%, 100% ethanol and xylene, followed by mounting with Pertex® mounting medium (Medite, Burgdorf, Germany) and examination under a bright field microscope.

### Histological scoring of colitis

H&E stained mouse intestinal sections were scored microscopically in a blinded manner by RW including assessments of crypt architecture (0-normal to 5-large ulcers), crypt abscesses (0-none to 3->10 crypt abscesses), tissue damage (0-none to 3-extensive mucosal damage), goblet cell depletion (0-normal to 3->50% depletion), inflammatory cell infiltration (0-occasional infiltration to 3-transmural infiltration) and local neutrophil counts (0-<5 to 3->20 in one field under 40X lenses). Crypt length (0–4) was measured using CellSens software (Olympus). This scoring system determines a combined score of histological colitis (maximum of 25) via the assessment of the above-mentioned parameters which reflect tissue damage and colitis severity.^6,28,29^

### Immunohistochemistry and immunofluorescence staining

Different antigen retrieval methods were used for different antigens. For Muc2, Grp78, and Ki67 staining, slides were boiled in citric acid buffer (10 mM citric acid and 0.05% Tween-20, pH 6.0) for 20 min in a microwave oven. For lysozyme staining, slides were boiled in Tris-EDTA buffer (10 mM Tris Base; 1 mM EDTA; 0.05% Tween-20, pH 9.0) for 20 min in a microwave oven. After antigen retrieval steps, the endogenous hydrogen peroxidase activity was quenched by incubating slides with 3% hydrogen peroxidase in PBS for 10 min. Sections were then blocked with 10% KPL blocking solution (KPL Inc, USA) in PBS for 1 h at room temperature (RT). Each primary antibody was titrated first to define the optimal staining concentration. For mature Muc2, 1 in 500 diluted rabbit anti-mouse Muc2 antibody (Muc2.3, in-house, against peptide NGLKPVRVPDADNC, previously described in^6^) was incubated with slides overnight at 4°C. For Grp78 staining, rabbit anti-mouse Grp78 (clone: GL-19, Sigma) antibody was diluted 1 in 100 in PBS with 5% KPL and incubated with sections overnight at 4°C. For lysozyme, rabbit anti-lysozyme antibody (Dako, USA) was diluted 1 in 500 in PBS with 5% FCS and applied with sections overnight at 4°C. For Ki-67, 1 in 200 diluted rabbit anti-human/mouse Ki-67 antibody (SP6, Thermo Fisher, MA5-14520) was diluted in 5% KPL in PBS and applied with sections for 2 h at RT. For detection of Muc2 maturation, 20 µg/ml FITC-conjugated Dolichos biflorus agglutinin (DBA) lectin (Sigma) was applied with sections for 30 min at RT after Muc2 primary antibody staining. After incubation with primary antibody, slides were washed 3 times in PBS. HRP-conjugated secondary antibodies against the species primary antibody raised in were incubated with sections for 2 h at room temperature. Unbound antibodies were washed with PBS and signal was detected using diaminobenzidine solution (Dako, CA, US). After counter-staining with hematoxylin, sections were dehydrated in an ethanol gradient and cleared in xylene before mounting with Pertex® mounting medium (Medite, Belgium). For Muc2 staining in differentiated organoids, BME-embedded organoids were dislodged with a spatula and solubilized in 5 mM EDTA in PBS for 1 h at 4°C on a rotating platform. Organoids were gently pelleted at 100 g for 5 min before fixed in 2% paraformaldehyde (PFA) in 1X PBS for 30 minutes at room temperature. Fixed organoids were washed extensively in PBS for 3 times at 100 g for 5 min before resuspended in 50 µl of 2% agarose. Once solidified, the agarose plug containing organoids are gently removed from the tube and then paraffin-embedded and sectioned at 10 µm thickness on super frost plus slides for staining. Organoids are permeabilized in PBS with 3% bovine serum albumin, 1% Triton X-100, and 1% Saponin for 30 mins. Mucin was reduced with 50 mM DTT in 0.1 M Tris-HCl (pH8) for 30 min, followed by alkylation using 25 mM iodoacetamide in 0.1 M Tris-HCl (pH8) in the dark at RT for 30 min. 1 in 500 diluted rabbit anti-mouse Muc2 antibody (Muc2.3) was incubated with organoids overnight at 4°C. Then, organoids were stained with anti-rabbit Alexa Flour-texas red secondary antibody (Invitrogen) and 20 µg/ml FITC-conjugated *Dolichos biflorus* lectin (Sigma). Organoids were stained with DAPI before imaged using the Olympus FV1200 Confocal Microscope.

### Alcian blue and periodic acid Schiff’s (PAS) staining

Alcian blue and PAS staining was used to assess the goblet cell mucin storage. After de-waxing in xylene and re-hydrating through ethanol gradients, paraffin sections were placed in Alcian blue solution (1% Alcian blue in 3% acetic acid, pH 2.5) for 10 min and washed under running tap water. Then, slides were treated with freshly prepared 1% periodic acid for 5 min and washed with dH_2_O before staining in Schiff’s reagent (Sigma) for 10 min. Slides were washed under running tap water for 5 min and then counterstained with hematoxylin (Gill’s Formula No.2, Sigma) for 30 s, dehydrated, cleared, and mounted with Pertex® mounting medium (Medite, Belgium).

### Morphometric assessments

To quantify positive Muc2 staining in tissue sections after immunohistochemistry staining, multiple pictures from areas with longitudinally sectioned crypts were taken randomly using an Olympus bright field microscope with a 40× lens to keep sampling process unbiased. Similarly, to quantify non-glycosylated Muc2 staining in tissue sections after immunofluorescent staining, multiple pictures from areas with longitudinally sectioned crypts were taken randomly using an Olympus FV1200 confocal microscopy with a 40× lens. In each image, positive staining was defined by RGB pixel picking tool in NIS-Elements software v.3.0 (Nikon, Tokyo, Japan). The percentage of the longitudinally sectioned crypt area occupied by positive staining was calculated for multiple crypts within each specimen and the average for that specimen determined and used for statistical analysis. For Ki-67 positive cell quantification, the number of positive staining nuclei per every 10 longitudinally cut crypts was counted.

### Colonic organoid culture and treatments

For *WT* and *Winnie* (n = 4) colonic organoid culture, whole colon was dissected and opened longitudinally in cold PBS, cut into 10 mm pieces, and incubated in EDTA solution for 1 h at room temperature, then digested with collagenase type I (2 mg/mL) supplemented with gentamicin (50 µg/mL) for 15–20 min at 37°C for 15 min. Then, colonic epithelia containing crypts were released from the tissue after vigorous pipetting. The isolated crypts were washed with DMEM/F12 medium and centrifuged at 50 x g for 5 min at 4°C and cells were embedded in Basement Membrane Extract (Invitrogen) in a 1:1 ratio. Then, 20 µL of the mixture was plated in a 24 well tissue culture plate and cultured in 50% L-WRN conditioned medium (a kind gift from Stappenbeck Lab) together with Y27632 at 10 µM (Rho-associated protein kinase inhibitor) and SB431542 at 10 µM (the transforming growth factor (TGF)-β type I receptor inhibitor). The crypts were expanded by serial culture until sufficient numbers were obtained for experimentation. To stimulate mucin production, organoids were differentiated in 5% L-WRN conditioned medium +95% of advanced DMEM supplemented with 20% FBS, 1% L-glutamine and 1% Penicillin/Streptomycin and SB431542 at 10 µM in presence of DAPT at 5 µM for 48 h. Organoids were harvested in TRIzol for RNA isolation and qPCR to look at gene expressions.

### Epithelial isolation and protein extraction for ELISA

Conventionally raised *WT* and *Winnie* (n = 4) were used for colonic epithelial (crypts) isolation. The same protocol was used as above for crypt isolation. The isolated crypts were lysed in 50 µL of cold RIPA buffer (50 mM Tris-HCI, pH 7.5, 150 mM NaCI, 1.0% Nonidet *P*-40, 0.1% sodium deoxycholate supplemented with Complete protease inhibitors and phosphate stop cocktail) for 15 min on ice and vortexed every 5 min. The lysates were centrifuged at 16,000 g for 20 min at 4°C to remove cell debris and stored at −80°C for further analysis. BCA assay was conducted to determine the protein concentration. 50 µL of the epithelial cell lysate was used for ELISA to measure Mip2a concentration. Final concentration was assessed by ELISA and then normalized to the total amount of protein determined by BCA assay.

### RNA-sequencing

To determine the transcriptome changes in differentiated *Winnie* compared to *WT* organoids. Organoids from 2 *WT* and 2 *Winnie* mice were established as above. After 48 h differentiation with DAPT at 20 µM, organoids were harvested and total mRNA was extracted using the TRizol/Chloroform method in combination with RNA column (RNA mini column, Bio-line). The RNA library preparation and pair-end sequencing was done by Australian Genome Research Facility (AGRF) using a HiSeq 2500 instrument (Illumina, San Diego, CA) with a maximal read length of 100 bp. The differential gene expression was analyzed through edgeR (version 3.22.3) using R (The R Foundation; version 3.5.0). Raw gene counts per million were used for transcriptional comparison. The gene ontology analyses were done using David Pathway online tool to identify the pathways.

## Supplementary Material

Supplemental Material
