# Supplementary material for: Gut microbiota shape the inflammatory response in mice with an epithelial defect
Source: Gut Microbes. 2021 Feb 28;13(1):1887720. doi: 10.1080/19490976.2021.1887720 (PMC7928202; doi:10.1080/19490976.2021.1887720)
Supplement: Supplemental Material [file KGMI_A_1887720_SM5617.zip › Supplementary information/Supplemental figure legends and table.docx]

**Supplemental figure 1**. A, Representative immunohistochemistry Ki67 stain in small intestine and distal colon (B) of mice described in Fig 1. Red arrows indicate Ki67 positive epithelial cells. Quantification of Ki67 positive cells in small intestine (C) and distal colon (D) showing intestinal epithelial cell proliferation in mice housed under GF and conventional conditions. Data points represent Ki67 positive cells per every 10 crypts counted. E, Representative immunohistochemistry of lysozyme in the small intestine of mice in Fig 1. F, Quantification of positive lysozyme staining per crypts of mice in E. Data points represent percent of positive staining per crypt quantified using VioPharm software. G, mRNA expression of *Reg3b and Reg3g* in the distal colon*.* Values are expressed as mean ± SEM and individual data points, n = 10 to 14. E, One-way ANOVA with Bonferroni's multiple comparison test; *compared to *WT* control, #compared to GF *WT*, and $compared to *Win* (**P < 0.01, ***P < 0.001). Scale bar = 200 µm.

**Supplemental figure 2**. Muc2 misfolding was improved in the small intestine and proximal colon of GF *Winnie* compared to *Winnie* mice. Immunofluorescent staining of Muc2 core peptide (Red) and DBA lectin for O-glycans (Green) showing non-glycosylated Muc2 (misfolded Muc2 in Red) accumulating in the small intestine and proximal colon of *Winnie* mice raised in conventional conditions and reducing in germ-free conditions.

**Supplemental figure 3**. A, Bright-field pictures of cultured colonic organoids isolated from *WT* mice and *Winnie* mice. B, *Muc2* expression in DAPT-treated *WT* and *Winnie* organoids confirming differentiation. n=8 per group, mixed gender. Mann-Whitney test (*P < 0.05). In B, *compared to *WT* PBS control, $compared to *Win* control (**P < 0.01; ****P <0.0001).

**Supplemental figure 4.** A, Smearplot showing differentially regulated genes in differentiated *Winnie* and *WT* organoids assessed by RNA-Seq. B, Full gene ontology report by DAVID Bioinformatics Tool with the input of 119 mostly differentially expressed genes between *WT* and *Winnie* organoids. C, Gene expression of *Ccr4*, *Tff2* and *Tnfrsf8* in differentiated *WT* and *Winnie* organoids. Mann-Whitney test.

**Supplemental figure 5.** A, Immunofluorescent staining of Muc2 core peptide (Red) and DBA lectin for O-glycans (Green) showing non-glycosylated Muc2 (misfolded Muc2 in Red) accumulating in the differentiated organoids isolated from *Winnie* compare to *WT* (low magnification for picture in **Fig 5A**). Scale bar in A = 20 µm.

Supplemental Table 1. Primer sequences.

| Gene name | Forward Primer 5’-3’ | Reverse Primer 5’-3’ |
| --- | --- | --- |
| *Il17a* | CTCCAGAAGGCCCTCAGACTAC | AGCTTTCCCTCCGCATTGACACAG |
| *Ifng* | AGCTCTTCCTCATGGCTGTTTC | ATGTTGTTGCTGATGGCCTGA |
| *Il1b* | CAACCAACAAGTGATATTCTCCATG | GATCCACACTCTCCAGCTGCA |
| *Mip2a* | GAAATCGTGCGTGACATCAAA | CACAGGATTCCATACCCAAGA |
| *Grp78* | TGCTGCTAGGCCTGCTCCGA | CGACCACCGTGCCCACATCC |
| *sXbp1* | GAGTCCGCAGCAGGTGC | CAAAAGGATATCAGACTCAGAATCTGA |
| *Muc2* | CCATTGAGTTTGGGAACATGC | TTCGGCTCGGTGTTCAGAG |
| *Atoh1* | GCTTCCTCTGGGGGTTACTC | GAAGGCGACAGGTCCTTCTG |
| *Hes1* | GGTCCTAACGCAGTGTCACC | GAGAGGTGGGCTAGGGACTT |
| *Spdef* | AAAGCCACTTCTGCACGTTACCAG | GTTGCCTGCTACTGTTCCCAGATG |
| *Agr2* | CGGTGAGGGCAGACATCACTGGA | CCGGTGCGCAGTTGGCTCTA |
| *Ccl1* | ACCATGAAACCCACTGCCAT | GTAAGCATGCTCTTGCTGTCAA |
| *Ccl3* | CGTTCCTCAACCCCCATC | TGTCAGTTCATGACTTTGTCATCAT |
| *Cxcl9* | TGGAGTTCGAGGAACCCTAGT | TTGTAGTGGATCGTGCCTCG |
| *Nos2* | CAGCTGGGCTGTACAAACCTT | CATTGGAAGTGAAGCGTTTCG |
| *Il23p19* | AGCGGGACATATGAATCTACTAAGAGA | GTCCTAGTAGGGAGGTGTGAAGTTG |
| *Nov* | CTGCATTGAACAGACCACAGA | TCTTGAACTGCAGGTGGATG |
| *Tff2* | TGCTCTGGTAGAGGGCGAG | CGACGCTAGAGTCAAAGCAG |
| *Calcrl* | AGGCGTGAACCAAACAGACTT | GTCCCAGGTCCTATTGCAGT |
| *Nt5e* | AACCCCTTTCCTCTCAAATCCA | CAGGGCGATGATCTTATTCACAT |
| *Ccr4* | ATCCTGAAGGACTTCAAGCTCCA | AGGTCTGTGCAAGATCGTTTCATGG |
| *Enpp3* | CAGTTGACAATGCCTTTGGAATG | CACTCTATCACAGGAGGTCTGG |
| *Slpi* | AGCCACAATGCCGTACTGACT | AGGCTTCCTCCACACTGGTT |
| *Tnfrsf8* | ACTACGTCAATGAAGACGGGA | TCACAGATTCGAGGAGAGTTCC |
| *Cma1* | CGCCCCTACATGGCCTATC | AGGAGGACTGTTATAGACCTTCC |
| *Cxcl11* | GGCTTCCTTATGTTCAAACAGGG | GCCGTTACTCGGGTAAATTACA |
