# Supplementary material for: Gut microbiota shape the inflammatory response in mice with an epithelial defect
Source: Gut Microbes. 2021 Feb 28;13(1):1887720. doi: 10.1080/19490976.2021.1887720 (PMC7928202; doi:10.1080/19490976.2021.1887720)
Supplement: Supplemental Material [file KGMI_A_1887720_SM5617.zip › Supplementary information/Supplementary figures.pdf]

Supplementary Materials

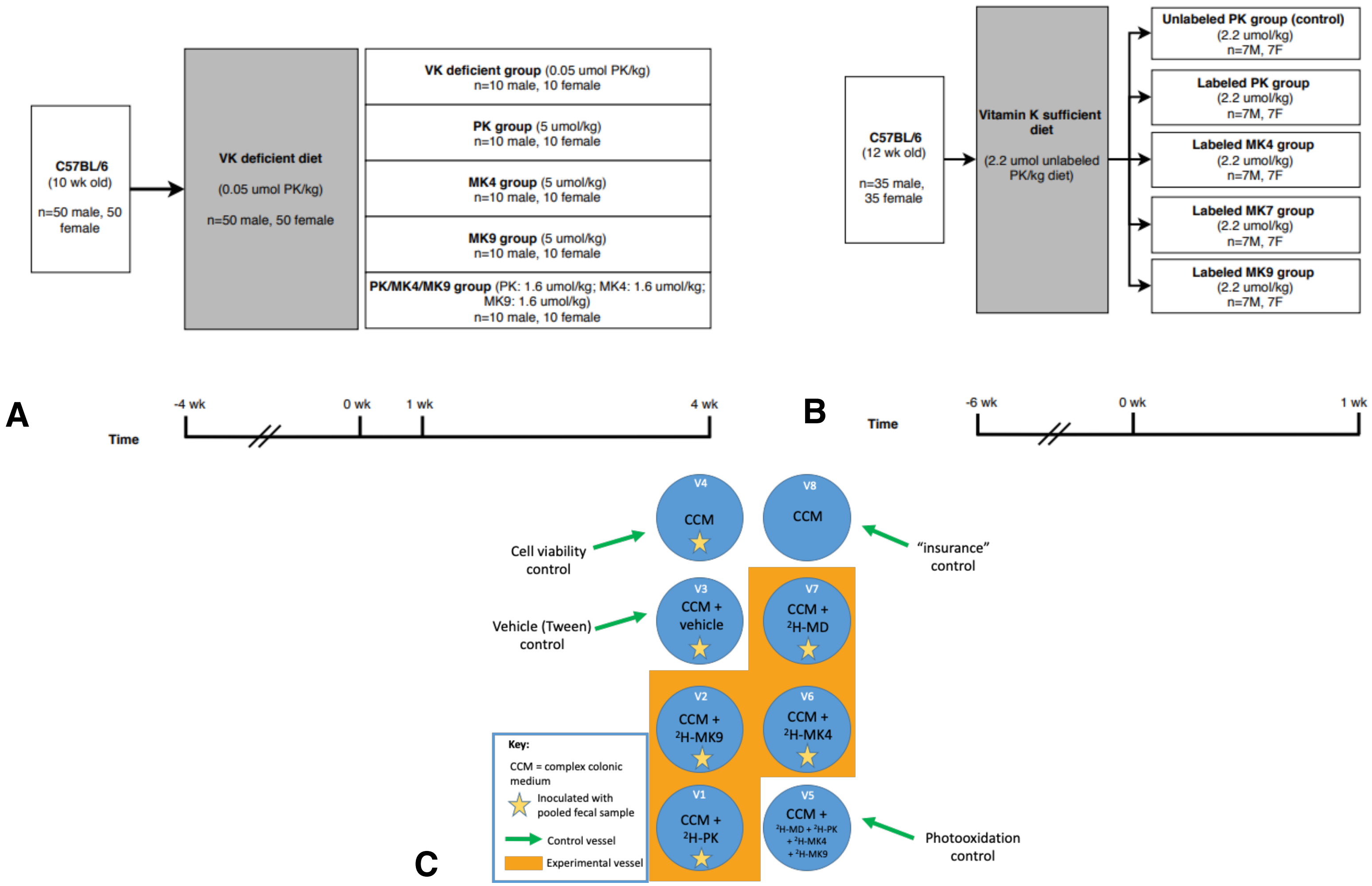

Supplemental Figure 1. Study designs of **A**) unlabeled vitamin K supplementation study (Study 1), **B**) stable isotope-labeled vitamin K supplementation study (Study 2), and **C**) *in vitro* fermentation study (Study 3). For Studies 1 and 2, cecal contents and feces were collected at sacrifice, and for Study 3 culture aliquots were collected at 0, 5, 10, 24, and 48h.

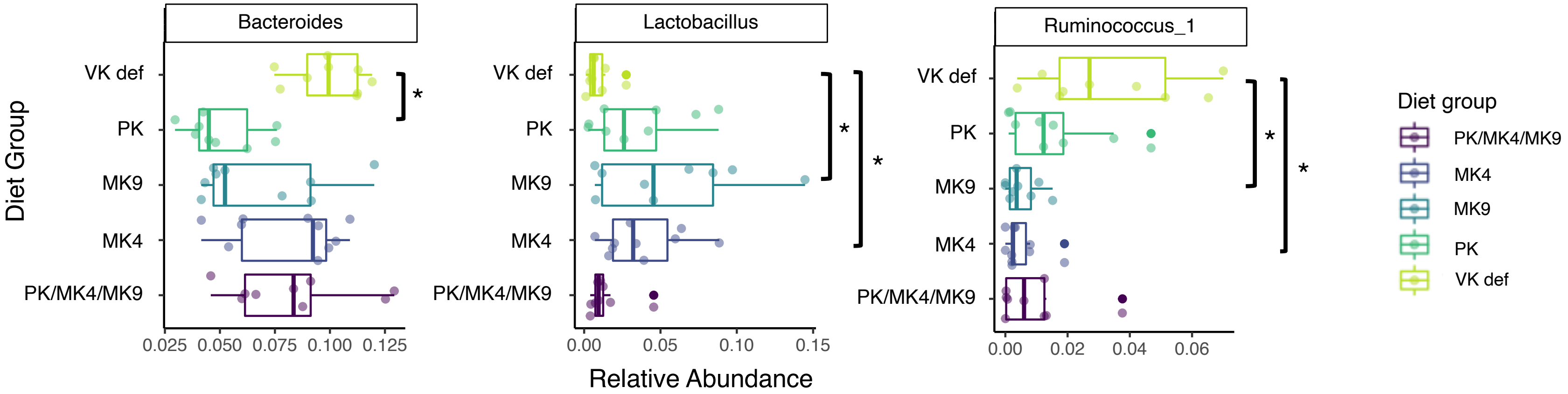

Supplemental Figure 2. Microbial genera that were significantly enriched across different diet groups in female mice in Study 1 (FDR-corrected p $\leqslant 0.1$). Assessed with Kruskal-Wallis tests across diet groups for all ASVs with a mean abundance threshold of 0.001 (the minimum mean value needed in at least one of the factor levels for an ASV to be retained in the analysis). *indicates a pairwise comparison p<0.05. No genera were significantly enriched across diet groups in male mice.

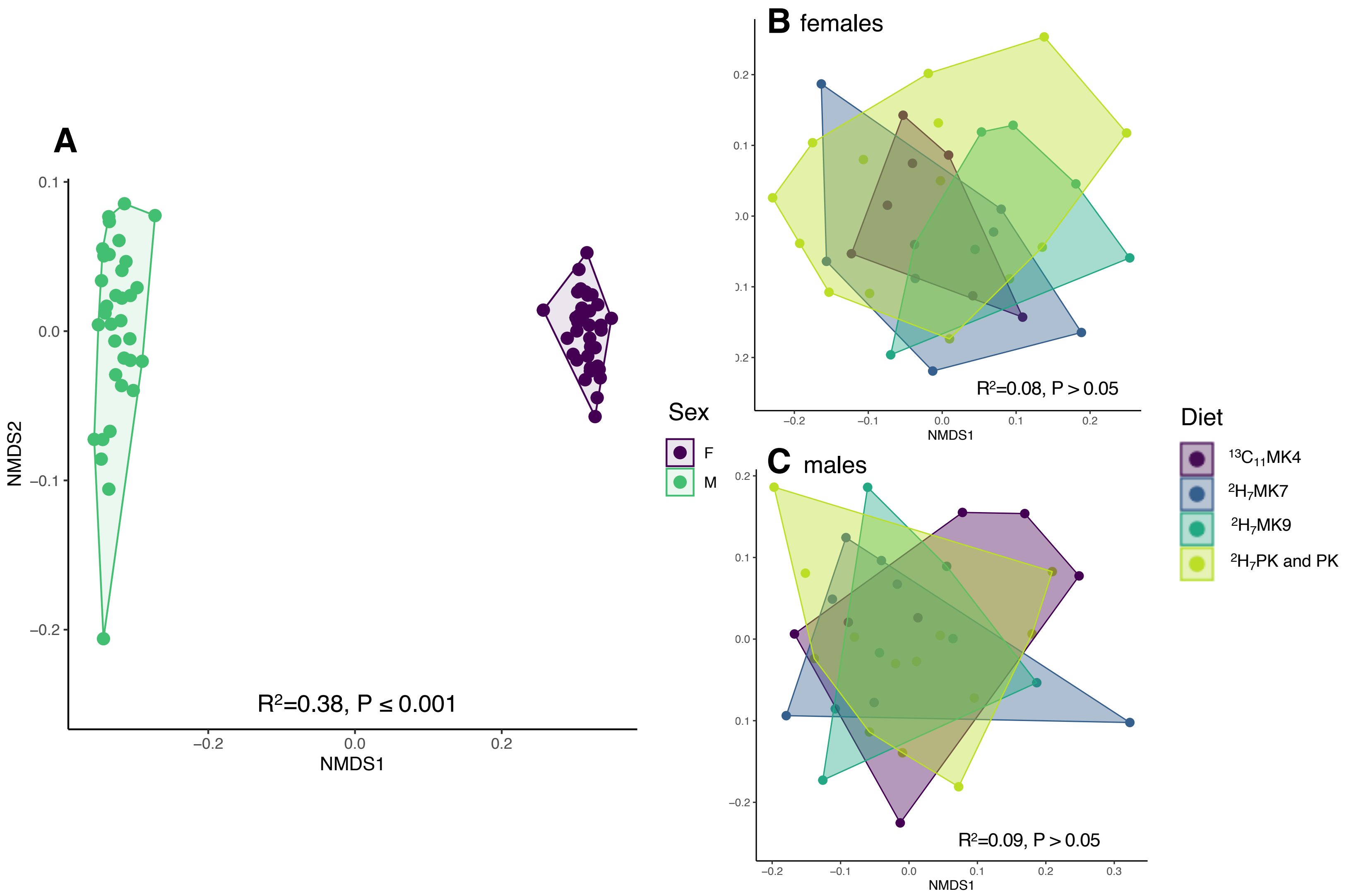

Supplemental Figure 3. Non-metric multidimensional scaling (NMDS) ordination of mouse cecal microbial communities in Study 2. Microbial communities of female and male mice were significantly different by (**A**) sex, but not by diet group in either (**B**) female or (**C**) male mice. The control (unlabeled PK) group and $^{2}H_{7}$PK groups were not significantly different and were combined in the diet panels above.

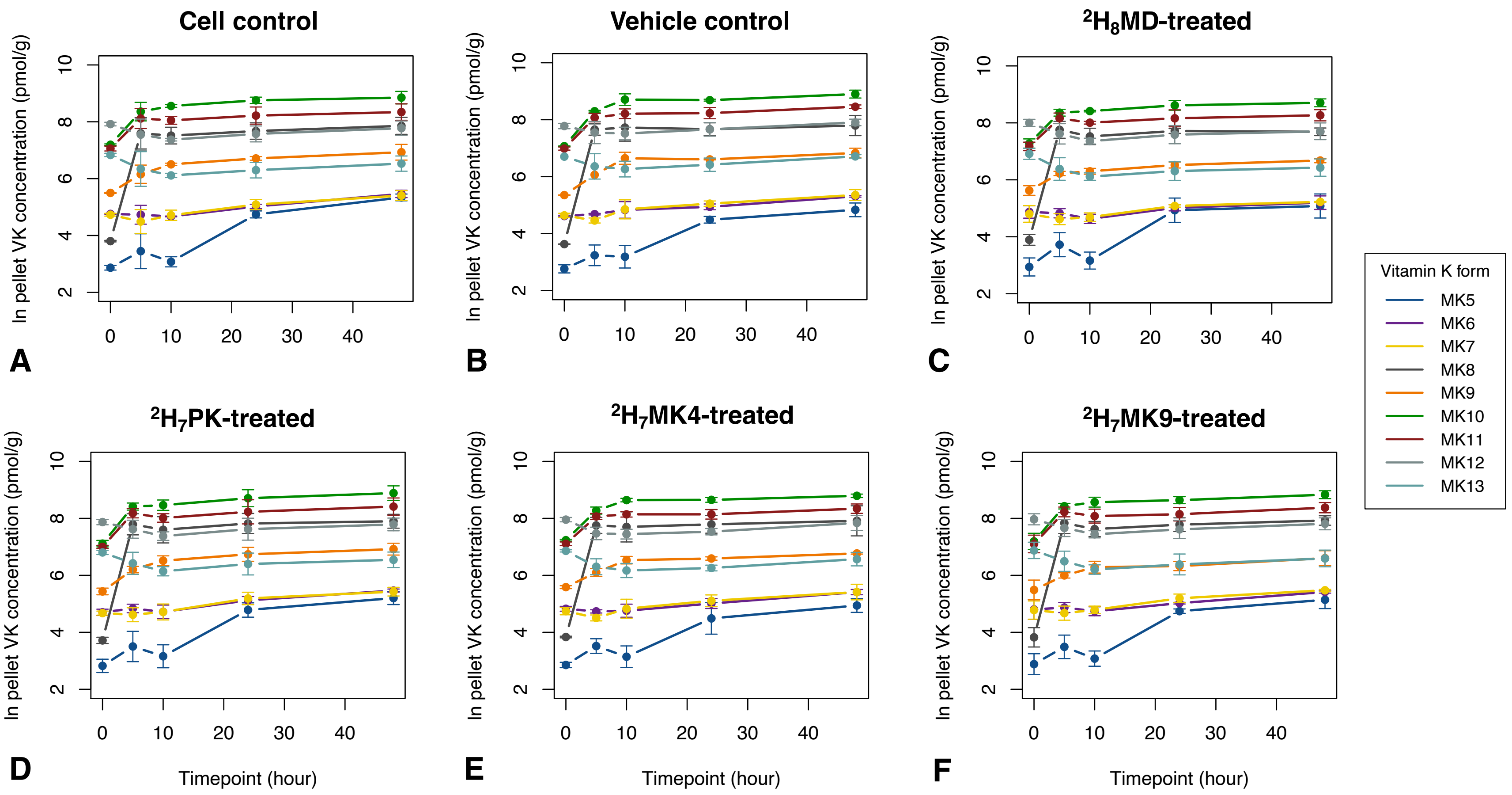

Supplemental Figure 4. Endogenous production of menaquinones did not statistically differ by vessel (controls or treated with ^{2}H-labeled vitamin K quinones) in either the pellet or supernatant fractions in Study 3. Menaquinone concentrations over time in the pellet fraction are shown in all inoculated vessels (**A-F**, supernatant data not shown). Points represent the mean of the natural log (ln) concentration from n=3 experiments, and bars indicate standard deviation.

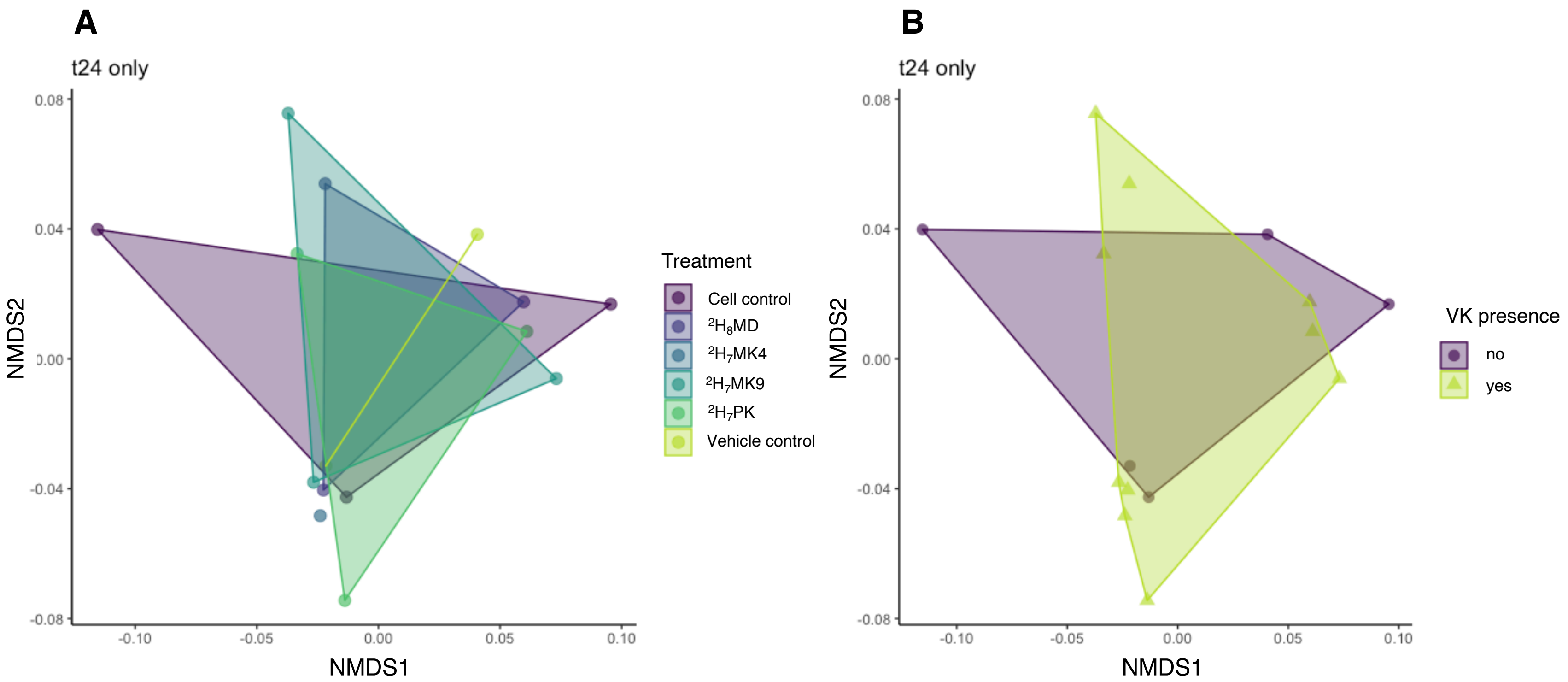

Supplemental Figure 5. Non-metric multidimensional scaling (NMDS) ordination of microbial communities in Study 3 based on Bray-Curtis dissimilarities, based on ^{2}H-vitamin K presence at t=24h only. Microbial community composition was not significantly different by (**A**) treatment vessel or (**B**) ^{2}H-vitamin K yes/no.

Supplemental Table 1. Microbial genera enriched by sex in Study 1. Assessed with Kruskal-Wallis tests across diet groups for all ASVs with a mean abundance threshold of 0.001.

	pvals	pvalsBon	pvalsFDR	F	M	taxonomy
1	5.22E-09	2.66E-07	2.66E-07	0.00116185	0.0023832	Bacteria; Firmicutes; Clostridia; Clostridiales; Ruminococcaceae; Ruminococcaceae_NK4A214_group
2	7.85E-09	4.00E-07	2.00E-07	0.00647757	0.00094817	Bacteria; Firmicutes; Bacilli; Lactobacillales; Aerococcaceae; Aerococcus
3	1.72E-07	8.76E-06	2.92E-06	0.03027347	0.00751472	Bacteria; Firmicutes; Bacilli; Lactobacillales; Lactobacillaceae; Lactobacillus
4	2.43E-06	0.00012409	3.10E-05	0.00787947	0.00269533	Bacteria; Firmicutes; Bacilli; Bacillales; Family_XII; Exiguobacterium
5	1.19E-05	0.00060491	0.00012098	0.00130204	0.00045936	Bacteria; Firmicutes; Clostridia; Clostridiales; Lachnospiraceae; Tyzzerella_3
6	1.71E-05	0.00087442	0.00014574	0.01365033	0.04224578	Bacteria; Firmicutes; Clostridia; Clostridiales; Ruminococcaceae; Ruminococcus_1
7	3.12E-05	0.00159094	0.00022728	0.0058688	0.00291127	Bacteria; Firmicutes; Clostridia; Clostridiales; Lachnospiraceae; Lachnospiraceae_FCS020_group
8	6.74E-05	0.00343753	0.00042969	0.00111768	0.00020024	Bacteria; Proteobacteria; Gammaproteobacteria; Pseudomonadales; Moraxellaceae; Acinetobacter
9	0.00020004	0.01020181	0.00113353	0.09976571	0.13420691	Bacteria; Firmicutes; Clostridia; Clostridiales; Lachnospiraceae; Lachnospiraceae_NK4A136_group
10	0.00020602	0.01050689	0.00105069	0.00822707	0.00487044	Bacteria; Firmicutes; Clostridia; Clostridiales; Lachnospiraceae; A2
11	0.00029037	0.01480882	0.00134626	0.0576087	0.04132901	Bacteria; Firmicutes; Clostridia; Clostridiales; Lachnospiraceae; Roseburia
12	0.00045907	0.02341266	0.00195105	0.00027462	0.00146447	Bacteria; Firmicutes; Clostridia; Clostridiales; Ruminococcaceae; Ruminiclostridium_6
13	0.00047354	0.02415072	0.00185775	0.00117145	0.00050844	Bacteria; Firmicutes; Clostridia; Clostridiales; Ruminococcaceae; Ruminococcaceae_UCG-013
14	0.00207597	0.10587456	0.00756247	0.00156898	0.00107578	Bacteria; Firmicutes; Clostridia; Clostridiales; Ruminococcaceae; Ruminococcaceae_UCG-009
15	0.0047076	0.24008783	0.01600586	0.00086419	0.00223204	Bacteria; Firmicutes; Clostridia; Clostridiales; Clostridiales_vadinBB60_group; NA
16	0.00572536	0.2919936	0.0182496	0.00411546	0.00582057	Bacteria; Bacteroidetes; Bacteroidia; Bacteroidales; Muribaculaceae; Muribaculum
17	0.00608833	0.31050482	0.01826499	0.00992664	0.00743227	Bacteria; Firmicutes; Clostridia; Clostridiales; Lachnospiraceae; GCA-900066575
18	0.00954349	0.48671807	0.02703989	0.02061761	0.0159521	Bacteria; Firmicutes; Clostridia; Clostridiales; Lachnospiraceae; Lachnoclostridium
19	0.0185654	0.94683541	0.04983344	0.0048625	0.00627405	Bacteria; Firmicutes; Clostridia; Clostridiales; Ruminococcaceae; Ruminococcaceae_UCG-003
20	0.02874637	1.46606476	0.07330324	0.11949416	0.10832548	Bacteria; Firmicutes; Clostridia; Clostridiales; Lachnospiraceae; NA
21	0.03889413	1.98360082	0.09445718	0.00235251	0.00281704	Bacteria; Firmicutes; Clostridia; Clostridiales; NA; NA

22	0.04485699	2.28770663	0.10398667	0.00153249	0.00201021	Bacteria; Firmicutes; Clostridia; Clostridiales; Ruminococcaceae; Anaerotruncus

Supplemental Table 2. Microbial genera enriched by sex in Study 2. Assessed with Kruskal-Wallis tests across diet groups for all ASVs with a mean abundance threshold of 0.001.

	pvals	pvalsBon	pvalsFDR	F	M	taxonomy
1	2.72E-14	1.22E-12	1.22E-12	0.04084387	0	Bacteria; Bacteroidetes; Bacteroidia; Bacteroidales; Marinifilaceae; Butyricimonas
2	7.25E-14	3.26E-12	1.63E-12	0	0.00382541	Bacteria; Tenericutes; Mollicutes; Anaeroplasmatales; Anaeroplasmataceae; Anaeroplasma
3	1.15E-13	5.17E-12	1.72E-12	0.00139146	1.59E-05	Bacteria; Bacteroidetes; Bacteroidia; NA; NA; NA
4	1.24E-10	5.60E-09	1.40E-09	0.00171409	0.00508379	Bacteria; Bacteroidetes; Bacteroidia; Bacteroidales; Rikenellaceae; Alistipes
5	3.35E-08	1.51E-06	3.01E-07	0.00080402	0.00466963	Bacteria; Firmicutes; Clostridia; Clostridiales; Lachnospiraceae; Butyrivibrio
6	4.24E-08	1.91E-06	3.18E-07	0.0049771	0.00042277	Bacteria; Cyanobacteria; Melainabacteria; Gastranaerophilales; NA; NA
7	4.56E-08	2.05E-06	2.93E-07	0.00147565	0.00010123	Bacteria; Firmicutes; Clostridia; Clostridiales; Ruminococcaceae; UBA1819
8	8.65E-08	3.89E-06	4.86E-07	0.04016903	0.08665802	Bacteria; Bacteroidetes; Bacteroidia; Bacteroidales; Tannerellaceae; Parabacteroides
9	1.13E-07	5.06E-06	5.63E-07	0.00746501	0.0173447	Bacteria; Firmicutes; Clostridia; Clostridiales; Ruminococcaceae; Ruminococcus_2
10	8.05E-07	3.62E-05	3.62E-06	0.00157398	0.00249625	Bacteria; Firmicutes; Clostridia; Clostridiales; NA; NA
11	1.17E-06	5.25E-05	4.77E-06	0.02908495	0.02201367	Bacteria; Firmicutes; Clostridia; Clostridiales; Ruminococcaceae; Ruminiclostridium_9
12	2.13E-06	9.57E-05	7.98E-06	0.05314524	0.03554222	Bacteria; Firmicutes; Clostridia; Clostridiales; Lachnospiraceae; Roseburia
13	6.01E-06	0.00027058	2.08E-05	0.00051995	0.00117832	Bacteria; Firmicutes; Erysipelotrichia; Erysipelotrichales; Erysipelotrichaceae; Erysipelatoclostridium
14	3.10E-05	0.00139325	9.95E-05	0.00152835	0.00081113	Bacteria; Firmicutes; Clostridia; Clostridiales; Ruminococcaceae; Ruminococcaceae_UCG-009
15	3.99E-05	0.00179404	0.0001196	0.00038562	0.00115252	Bacteria; Proteobacteria; Gammaproteobacteria; Enterobacteriales; Enterobacteriaceae; Escherichia/Shigella
16	4.72E-05	0.002123	0.00013269	0.00405097	0.00277147	Bacteria; Firmicutes; Clostridia; Clostridiales; Ruminococcaceae; Ruminiclostridium_5
17	5.51E-05	0.00247977	0.00014587	0.02559956	0.01525535	Bacteria; Firmicutes; Clostridia; Clostridiales; Ruminococcaceae; Intestinimonas
18	0.00020333	0.00914978	0.00050832	0.00864373	0.01618888	Bacteria; Firmicutes; Clostridia; Clostridiales; Lachnospiraceae; GCA-900066575
19	0.00030413	0.01368607	0.00072032	0.00048331	0.00111944	Bacteria; Firmicutes; Clostridia; Clostridiales; Ruminococcaceae; Ruminococcus_1
20	0.00045127	0.02030729	0.00101536	0.00155663	0.00286344	Bacteria; Verrucomicrobia; Verrucomicrobiae; Verrucomicrobiales; Akkermansiaceae; Akkermansia

21	0.00060938	0.02742202	0.00130581	0.0010129	0.00218926	Bacteria; Firmicutes; Clostridia; Clostridiales; Ruminococcaceae; Ruminococcaceae_UCG-013
22	0.00079284	0.03567759	0.00162171	0.00226039	0.00317439	Bacteria; Firmicutes; Clostridia; Clostridiales; Lachnospiraceae; Tyzzerella
23	0.00135217	0.06084778	0.00264556	0.08814692	0.06786638	Bacteria; Bacteroidetes; Bacteroidia; Bacteroidales; Bacteroidaceae; Bacteroides
24	0.00146834	0.06607539	0.00275314	0.01413243	0.00569975	Bacteria; Firmicutes; Clostridia; Clostridiales; Lachnospiraceae; Lachnospiraceae_FCS020_group
25	0.00229285	0.10317847	0.00412714	0.00234073	0.00361833	Bacteria; Firmicutes; Clostridia; Clostridiales; Lachnospiraceae; Lachnospiraceae_UCG-004
26	0.00248958	0.11203112	0.00430889	0.02757009	0.03868883	Bacteria; Firmicutes; Clostridia; Clostridiales; Ruminococcaceae; Ruminiclostridium
27	0.00253896	0.11425329	0.0042316	0.01657085	0.0330076	Bacteria; Firmicutes; Clostridia; Clostridiales; Lachnospiraceae; A2
28	0.00522657	0.23519582	0.00839985	0.00076867	0.00226468	Bacteria; Proteobacteria; Alphaproteobacteria; Rhodospirillales; NA; NA
29	0.00643805	0.28971206	0.00999007	0.02800328	0.0367126	Bacteria; Firmicutes; Clostridia; Clostridiales; Lachnospiraceae; Lachnoclostridium
30	0.01719459	0.77375677	0.02579189	0.01609204	0.01358876	Bacteria; Firmicutes; Clostridia; Clostridiales; Ruminococcaceae; Oscillibacter
31	0.02117567	0.95290496	0.03073887	0.00120571	0.00186375	Bacteria; Firmicutes; Clostridia; Clostridiales; Lachnospiraceae; Acetitomaculum
32	0.03073465	1.38305942	0.04322061	0.00963286	0.00691248	Bacteria; Firmicutes; Clostridia; Clostridiales; Ruminococcaceae; Ruminococcaceae_UCG-014

**Supplemental Table 3.** Microbial genera enriched by sex in Study 1 *with the VK deficient group removed*. Assessed with Kruskal-Wallis tests across diet groups for all ASVs with a mean abundance threshold of 0.001.						
	pvals	pvalsBon	pvalsFDR	F	M	taxonomy
1	1.38E-08	7.03E-07	7.03E-07	0.03547894	0.00710056	Bacteria; Firmicutes; Bacilli; Lactobacillales; Lactobacillaceae; Lactobacillus
2	7.13E-08	3.64E-06	1.82E-06	0.00115318	0.00244007	Bacteria; Firmicutes; Clostridia; Clostridiales; Ruminococcaceae; Ruminococcaceae_NK4A214_group
3	4.31E-07	2.20E-05	7.32E-06	0.00769984	0.00114125	Bacteria; Firmicutes; Bacilli; Lactobacillales; Aerococcaceae; Aerococcus
4	1.80E-06	9.19E-05	2.30E-05	0.00865963	0.04577166	Bacteria; Firmicutes; Clostridia; Clostridiales; Ruminococcaceae; Ruminococcus_1
5	1.21E-05	0.00061791	0.00012358	0.00921354	0.00323513	Bacteria; Firmicutes; Bacilli; Bacillales; Family_XII; Exiguobacterium
6	7.39E-05	0.00376939	0.00062823	0.00635087	0.0030871	Bacteria; Firmicutes; Clostridia; Clostridiales; Lachnospiraceae; Lachnospiraceae_FCS020_group
7	8.42E-05	0.00429493	0.00061356	0.00132986	0.00023159	Bacteria; Proteobacteria; Gammaproteobacteria; Pseudomonadales; Moraxellaceae; Acinetobacter
8	0.00011447	0.00583777	0.00072972	0.00126779	0.00045602	Bacteria; Firmicutes; Clostridia; Clostridiales; Lachnospiraceae; Tyzzerella_3
9	0.00014125	0.00720375	0.00080042	0.00891032	0.00510935	Bacteria; Firmicutes; Clostridia; Clostridiales; Lachnospiraceae; A2
10	0.00019122	0.0097524	0.00097524	0.01092064	0.00716025	Bacteria; Firmicutes; Clostridia; Clostridiales; Lachnospiraceae; GCA-900066575
11	0.00043528	0.02219951	0.00201814	0.0012272	0.00047751	Bacteria; Firmicutes; Clostridia; Clostridiales; Ruminococcaceae; Ruminococcaceae_UCG-013
12	0.00044209	0.02254669	0.00187889	0.06167749	0.04462325	Bacteria; Firmicutes; Clostridia; Clostridiales; Lachnospiraceae; Roseburia
13	0.00101428	0.05172834	0.0039791	0.07103429	0.09376134	Bacteria; Bacteroidetes; Bacteroidia; Bacteroidales; Bacteroidaceae; Bacteroides
14	0.00131967	0.06730328	0.00480738	0.00381052	0.00597125	Bacteria; Bacteroidetes; Bacteroidia; Bacteroidales; Muribaculaceae; Muribaculum
15	0.00222228	0.11333604	0.00755574	0.00010744	0.00144685	Bacteria; Firmicutes; Clostridia; Clostridiales; Ruminococcaceae; Ruminiclostridium_6
16	0.00262596	0.13392378	0.00837024	0.00160682	0.00102665	Bacteria; Firmicutes; Clostridia; Clostridiales; Ruminococcaceae; Ruminococcaceae_UCG-009
17	0.0048593	0.24782405	0.01457789	0.09946758	0.12988731	Bacteria; Firmicutes; Clostridia; Clostridiales; Lachnospiraceae; Lachnospiraceae_NK4A136_group
18	0.0062324	0.31785231	0.01765846	0.02217314	0.01665314	Bacteria; Firmicutes; Clostridia; Clostridiales; Lachnospiraceae; Lachnoclostridium
19	0.00835615	0.4261637	0.02242967	0.00131793	0.00069955	Bacteria; Firmicutes; Clostridia; Clostridiales; Lachnospiraceae; Dorea
20	0.01978487	1.00902845	0.05045142	0.00412091	0.00589963	Bacteria; Bacteroidetes; Bacteroidia; Bacteroidales; Tannerellaceae; Parabacteroides

21	0.02010627	1.02542001	0.04882952	0.12402588	0.1104479	Bacteria; Firmicutes; Clostridia; Clostridiales; Lachnospiraceae; NA
22	0.02059843	1.05051984	0.0477509	0.00071388	0.00151132	Bacteria; Proteobacteria; Alphaproteobacteria; Rhodospirillales; NA; NA
23	0.02392794	1.22032508	0.05305761	0.00079267	0.00173097	Bacteria; Firmicutes; Clostridia; Clostridiales; Clostridiales_vadinBB60_group; NA
